# A simple plant–mycorrhizal fungal resource trade co‐evolution model explains mutualism stability, extinction and transitory parasitism via fitness feedback

**DOI:** 10.1111/nph.70540

**Published:** 2025-09-12

**Authors:** Sally V. Grasso, Megan H. Ryan, Felipe E. Albornoz, Michael Renton

**Affiliations:** ^1^ School of Biological Sciences University of Western Australia Perth WA 6009 Australia; ^2^ UWA School of Agriculture and Environment University of Western Australia Perth WA 6009 Australia; ^3^ Commonwealth Scientific and Industrial Research Organisation (CSIRO), Environment GPO Box 7229 Waterford WA 6152 Australia

**Keywords:** co‐evolution, modelling, mutualism, mycorrhizas, nutrient exchange, parasitism

## Abstract

- The mutualism between mycorrhizal fungi and plants has persisted for over 400 million years, despite the mutualism paradox predicting that mutualisms should be evolutionarily unstable due to the fitness advantages of cheating. It is widely accepted that mutual benefit alone is not sufficient for stable mutualism, and so a search for additional stabilising mechanisms has been the focus of past investigation. In this work, we test the assumption that cheating is an omnipresent threat to mutualism; hence, additional mechanisms for stability are needed.
- We developed a novel individual‐based model of a plant and mycorrhizal fungus, where mechanisms commonly thought necessary for mutualism stability, for example, partner choice, are absent. The organisms take up carbon and phosphorus at different uptake efficiencies, exchange resources, and grow depending on their limiting resource over 10 time steps. We simulated co‐evolution of resource trading strategies over 2000 generations, under 231 nutrient uptake efficiency combinations.
- Evolutionarily stable mutualism evolved in 66% of the nutrient uptake efficiency combinations tested. Parasitism and Darwinian extinction also occurred.
- Our results suggest that different nutrient uptake efficiencies, Leibig's law of the minimum, and one‐to‐one resource trade are sufficient to explain stable mutualism, parasitism and Darwinian extinction, via fitness feedback, without additional mechanisms.

## Introduction

Most terrestrial plants form physical associations in their roots with one or more types of mycorrhizal fungi. The main mycorrhizal fungi include arbuscular mycorrhizal fungi (AMF), ectomycorrhizal fungi (EMF), ericoid mycorrhizal fungi (ErMF), and orchid mycorrhizal fungi (OMF) (Brundrett & Tedersoo, 2018). The most well‐known function of these associations is resource trade, where the plant host provides carbon (C) to the fungus and receives phosphorus (P), nitrogen (N), and/or other minerals in exchange (Genre *et al*., 2020). However, this trade does not always benefit all parties involved, and interactions between plants and mycorrhizal fungi span the parasitism to mutualism continuum (Johnson *et al*., 1997; Merckx *et al*., 2009; Brundrett & Tedersoo, 2018; Strullu‐Derrien *et al*., 2018; Albornoz *et al*., 2021; Zhou *et al*., 2021). Although much is known about plant–mycorrhizal associations, an important question regarding their evolution remains unanswered: ‘Why has the mutualistic partnership not collapsed under the exploitation of parasitic strains of plants and fungi?’

There are examples of breakdowns of the trade‐based mutualism between plants and mycorrhizal fungi across evolutionary time. It is not uncommon for plant lineages to have lost, gained, and regained the ability to form associations with different mycorrhizal types over their evolutionary history (Maherali *et al*., 2016; Brundrett & Tedersoo, 2018). For instance, during the Jurassic and Late Cretaceous period, several plant lineages lost their ability to form associations with their original mycorrhizal partner, the ancestors of AMF, but gained the ability to partner with the newly evolved EMF, ErMF, or OMF (Brundrett & Tedersoo, 2018). Yet many plant lineages have retained the mutualism with AMF formed over 400 million years ago (Maherali *et al*., 2016; Brundrett & Tedersoo, 2018). This brings forth questions such as ‘Why did some plants lose their association with AMF while others did not?’ and ‘Why did some plants gain associations with new types of mycorrhizal fungi while others abandoned the trade mutualism entirely?’

To better understand the evolution of mycorrhizal associations, we can look at present day interactions as well as at other types of mutualisms. In the study of plant–mycorrhizal co‐evolution, and of the evolution of cooperation in general, it is recognised that cheating organisms should have a fitness advantage. That is: a partner in a mutualistic exchange who ‘cheats’ by receiving benefits from their partner but does not provide fitness benefits to them in return, should have a fitness advantage over an ‘honest’ partner who does. Cheater organisms should reproduce at a higher rate than honest organisms, eventually take over the population, and cause the extinction of cooperative phenotypes, ultimately causing the mutualism to breakdown at evolutionary scales. This is known as the mutualism paradox (Heath & Stinchcombe, 2014; Maherali *et al*., 2016). This prediction of mutualism instability is in stark contrast to the long‐lasting stable mutualisms we observe in the present day (Frederickson, 2017). The paradox is that we still observe persistent mutualisms despite the ‘logical’ prediction that cheaters should cause them to collapse. So, the question becomes not, ‘Why do mutualisms breakdown?’, but ‘Why do mutualisms emerge at all, and how do they persist?’ (Sachs *et al*., 2004; Hammerstein & Noë, 2016).

According to the logic of the mutualism paradox, mutual benefit alone is not enough to prevent evolutionary pressures from causing mutualism breakdown. It is argued, therefore, that for mutualisms to persist there must be stabilising mechanisms that prevent cheaters from causing mutualism breakdown (Axelrod & Hamilton, 1981; Axelrod & Dion, 1988; Bull & Rice, 1991; Sachs *et al*., 2004; Foster & Wenseleers, 2006; Leigh Jr., 2010; Nowak, 2012; Werner *et al*., 2014; Akçay, 2015; Hammerstein & Noë, 2016). Multiple stabilising mechanisms have been proposed, with several accepted as major contributors to mutualism stability. The transfer of symbionts between parent and offspring (vertical transmission), low variation within symbiont populations, restricted options for partners, and a population structure that leads to repeated interactions between individuals are thought to stabilise mutualisms in general (Herre *et al*., 1999). The roles of other possible stabilising mechanisms are still being actively investigated and debated.

Plant–mycorrhizal fungal interactions are different from other well‐studied mutualistic partnerships. Except for repeated interactions between individuals, plant–mycorrhizal interactions do not have most of the general mutualism stabilising mechanisms. Plant hosts source mycorrhizal partners from their environment, as opposed to inheriting them. There can be large variation in partner quality on both sides of the interaction (Johnson *et al*., 1997), and plants and fungi can both have multiple partners. Plant and mycorrhizal researchers are therefore searching for other stabilising mechanisms operating in plant–mycorrhizal fungal interactions. Several stabilising mechanisms have been proposed including partner choice, genetic correlation and dispersal, and specialisation (Frank, 1994; Schwartz & Hoeksema, 1998;Nowak, 2012; Werner *et al*., 2014; Akçay, 2015; Hammerstein & Noë, 2016). Partner choice, a term which often includes all forms of discrimination between potential and actual partners, has been put forward as essential for mutualism stability between plants and mycorrhizal fungi (Nowak, 2012; Werner *et al*., 2014; Akçay, 2015; Hammerstein & Noë, 2016). It has also been proposed as a major stabilising mechanism in other mutualisms (Akçay, 2015; Hammerstein & Noë, 2016). Several theoretical models have been created to test these proposed stabilising mechanisms in plant–mycorrhizal fungal mutualisms.

Most models that have been developed to explicitly investigate these proposed stabilising mechanisms in plant–mycorrhizal fungal interactions are based on biological market theory and resource competition models (Kummel & Salant, 2006; Cowden & Peterson, 2009; De Mazancourt & Schwartz, 2010; Wyatt *et al*., 2014; Bever, 2015; Bachelot & Lee, 2018; Christian & Bever, 2018; Steidinger & Peay, 2021). These models evaluate the effects of resource specialisation (De Mazancourt & Schwartz, 2010; Wyatt *et al*., 2014), accuracy of partner rewards (Bever, 2015; Christian & Bever, 2018), function type of partner rewards (Bachelot & Lee, 2018; Steidinger & Peay, 2021), and level of spatial control of partner rewards (Cowden & Peterson, 2009) on mutualism stability. These investigations show that proportionally and accurately rewarding honest mycorrhizal fungus partners has a stabilising effect on mutualism. They also demonstrate that resource specialisation can improve the focus organism's fitness under certain parameters.

Biological market models assume that the focus organism can optimise the quality of its partners for its own benefit through partner choice (Noe & Hammerstein, 1994). This is itself a proposed stabilising mechanism of mutualism. Also, many of the aforementioned models have the partner organisms fixed as being either beneficial or deleterious to the focus organism across the time span of the model. This means that the species cannot change from mutualistic to parasitic within these models. Except for Cowden & Peterson (2009), the models are executed at the population level. To the best of our knowledge, there are no models of plant–mycorrhizal fungal trade interactions that are executed at the individual level, contain repeated one‐to‐one interactions, and allow the interaction types to change from mutualism to parasitism throughout the timespan of the model.

Here we report on a novel individual‐based evolution model of resource trade strategies between a plant and mycorrhizal fungus which we have developed. Fitness is determined by one‐to‐one growth feedback and Leibig's law of the minimum. This allows us to exclude partner choice and most other proposed mutualism stabilising mechanisms, test the necessity of those mechanisms, and compare results to current models of plant‐mycorrhizal fungus evolution. We ask, ‘Given this biologically realistic model, will mutual benefit alone be enough for mutualistic trade between plants and fungi to emerge or will the lack of proposed mutualism stabilizing mechanisms prevent it?’

## Description

### The fitness model

We developed a novel fitness model based on a scenario where a plant and a mycorrhizal fungus obtain resources, exchange resources, and grow over a life cycle. We aimed to balance simplicity with representing the key biological processes involved in real plant–mycorrhizal fungus interactions. Thus, as in any abstract model, we have included only the processes most important to a simple trade‐based mutualism between a plant and mycorrhizal fungus rather than trying to include all processes of plant–mycorrhizal fungi interactions. We have restricted the resources exchanged and needed for growth to two elements, nominally C and P. The resulting model allows us to focus on the patterns of behaviour and broad qualitative features that arise across an unrestricted parameter space. The parameters and units of the model are defined relative to each other, rather than being real‐life values. Consequently, the parameter values used in this model do not necessarily need to take on the values of what we see in the real world to produce relevant results. Additionally, as this investigation is focussed on the evolution of plants and mycorrhizal fungi, we do not restrict ourselves to only the parameter values representing what we observe in the present day.

**Fig. 1:**
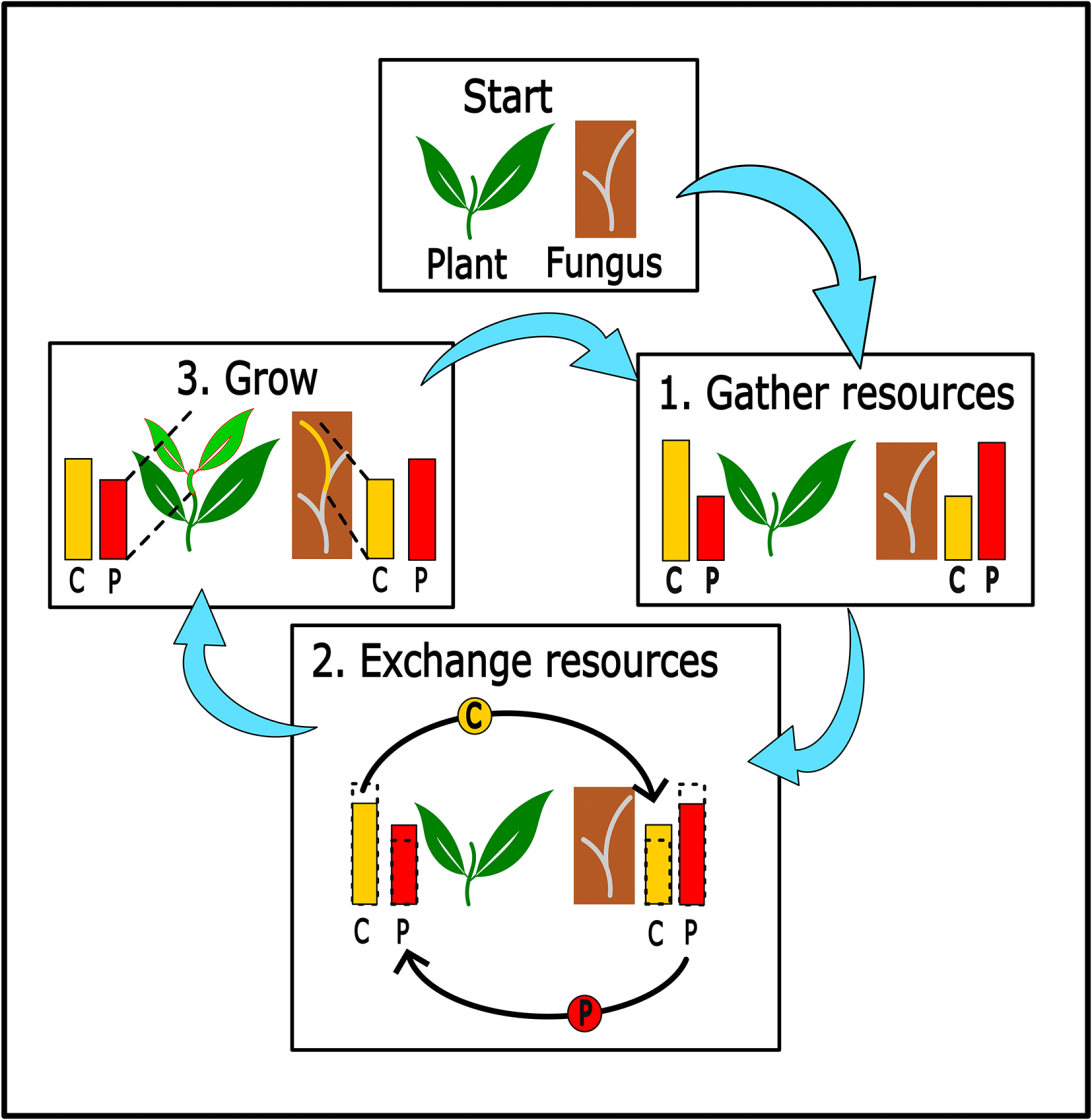
Schematic of one timestep of the fitness model. Each timestep has three stages: (1) gather resources, (2) exchange resources, and (3) grow. At the beginning of the timestep the plant and fungus both take up carbon (C) and phosphorus (P) from the environment from an unlimited source. The amount of each resource they acquire is equal to their biomass (initial biomass is 1) multiplied by their uptake efficiency for that resource. As the plant is specialised in gathering C and the fungus in gathering P their uptake efficiencies for these resources were always set to 100%, and so they take up an amount of these nutrients equal to their current biomass. The organisms are less efficient at taking up their nonspecialised resource, P for plant and C for fungus, and so the uptake of these nutrients are equal to their current biomass multiplied by their nonspecialised nutrient uptake efficiency, the plant P uptake efficiency and fungus C uptake efficiency. The fungus C uptake efficiency and the plant P uptake efficiency were set between 0 and 100% and remained constant. After gathering resources, the organisms transferred a percentage of their gathered specialised resource to their partner. The plant could give between 0 and 100% of its C to the fungus, and the fungus between 0 and 100% of its P to the plant. These parameters were called the ‘plant C to fungus’ and ‘fungus P to plant’, respectively. The organisms were also able to take their specialised resource from their partner. The plant could take a percent of C from the fungus, represented by a negative plant C to fungus percent. The fungus could do the same with P. Each organism was prevented from taking more of their specialised resource than their partner had gathered. The plant C to fungus and fungus P to plant remain constant throughout the 10 time steps of the life cycle. After exchanging resources, the plant and fungus increase in biomass equal to the amount of whichever resource they have least off, which could be either C or P. This allows either resource to become growth limiting. In the next timestep, the organisms gather resources based on their new biomasses. The final biomass of each organism is taken as their fitness value. The organisms do not store resources from previous time steps.

The model explicitly includes the uptake rates of C and P, the exchange of these resources, and biomass gains of each organism. This allows the interdependence of these variables to be captured in our model. The model simulates the nutrient uptake, exchange, and growth of a plant and mycorrhizal fungus over 10 time steps and returns a final fitness value for the plant and fungus. The mathematical relationship that arises from this fitness model is the pair of coupled recursive equations shown in Eqn 1, where *X*_*n*_ and *Y*_*n*_ are, respectively, the plant and fungus biomass at timestep *n*; *α* is the plant P uptake efficiency; *β* is the fungus C uptake efficiency; *γ* the percentage of plant C given to the fungus; and ε is the percentage of fungus P given to the plant. Together, the values *γ* and *ε* make the resource exchange strategy (*γ*, *ε*). The quantities *α* and *β* can take values anywhere between 0 and 1 inclusive, and γ and ε can take any values between −1 and 1 inclusive. The nutrient uptake efficiencies *α* and *β* represent efficiency relative to the size of the organism and thus can be seen as accounting for both the nutrient availability and the organism's ability to obtain it. The initial conditions at timestep *n* = 0 are *X*_0_ = *Y*_0_ = 1. These equations are coupled and recursive, where the growth of each organism is dependent on the other, and each time step builds on the one before. In instances where one organism ‘gives’ a negative amount, it may attempt to take more resource than the other organism has, which results in an interaction outside of the bounds of these two equations. In such a case, the first organism will take all the available resource from the second organism in that time step, but no more. This condition can change between time steps, as *X*_*n*_ and *Y*_*n*_ change. Additional equations describing the mathematical relationship outside of the range of Eqn 1 are in the Supporting Information Methods S1 (Eqns S1–S3). A schematic of a single time step of the growth model is shown in Fig. 1.

(Eqn 1) Xn+1=Xn+minαXn+εYn,Xn1−γYn+1=Yn+minYn1−ε,βYn+γXn

The different types of mycorrhizal fungi have different abilities to source their own C. Arbuscular mycorrhizal fungi have no ability to source their own C, receiving it entirely from their plant hosts (Genre *et al*., 2020). In our model, they are represented in scenarios where the fungus C uptake efficiency is 0%. Genetic analysis shows that modern AMF have remnants of genes related to breaking down plant cell walls, and so it is possible that their ancestors had some ability to source their own C (Morin *et al*., 2019). It is therefore still interesting to consider scenarios in which the fungus C uptake efficiency is not 0% when one is considering the history of AMF.

Other types of mycorrhizal fungi, such as EMF, OMF, and ErMF, have differing abilities to extract C from surrounding soil (Lindahl & Tunlid, 2015; Martino *et al*., 2018; Rasmussen, 2002; Hoysted *et al*., 2022). Ectomycorrhizal fungi have a very limited ability to acquire C from the soil; the majority of their metabolic C is provided by their plant hosts (Lindahl & Tunlid, 2015). EMF lineages differ in ability to break down soil organic matter, appearing to be at various stages along the evolutionary transition from a saprotrophic to purely mycorrhizal lifestyle (Miyauchi *et al*., 2020). While present‐day EMF are represented in scenarios where the fungus C uptake efficiency is low, their ancestors could be represented by scenarios where the fungus C uptake efficiency is greater. Present‐day OMF and ErMF have a greater ability to source C from the soil and have both biotrophic and saprotrophic capabilities (Genre *et al*., 2020). These fungi are represented by scenarios where the fungus C uptake efficiency is > 0%.

### Coupled fitness landscapes

Using the results from our novel fitness model we generated coupled fitness landscapes for combinations of plant P uptake efficiencies and fungus C uptake efficiencies. We used a total of 231 nutrient uptake efficiency combinations. There were 11 values for fungus C uptake efficiency from 0 to 100% in 10% increments, and 21 values for plant P uptake efficiency from 0 to 100% in 5% increments. For each nutrient uptake efficiency combination, we produced a plant fitness landscape and a fungus fitness landscape for 500 values for plant C to fungus and fungus P to plant between −100 and 100%. The plant and fungus fitness landscapes are dependent on each other and so are referred to as ‘coupled fitness landscapes’. On many coupled fitness landscapes there were areas where the biomass of one or both organisms did not increase past the initial value of 1. Organisms that had no growth could not go on to reproduce in the evolution simulations and so these areas were named ‘extinction zones’.

### Resources received landscapes

We calculated the total amount of C and P exchanged between the plant and the fungus over their lifetime and used it to produce the resources received landscapes for 21 nutrient uptake efficiency combinations. The values of fungus C uptake efficiency and plant P uptake efficiency were 0, 10, 20, 50, and 80%. The values for the plant C to fungus and fungus P to plant were those values for that particular combination of nutrient uptake efficiencies that resulted in greater than zero growth, which we call the ‘viable resource exchange range’. This viable resource exchange range was split into 500 increments and the total amount of C and P exchanged between the plant and fungus was calculated for each resource exchange strategy.

### Interaction type landscapes

Using the results from the coupled fitness landscapes, we produced an interaction type landscape for each of the 231 nutrient uptake efficiency combinations. We use the terms mutualism, parasitism, and competition in line with definitions used by Arthur & Mitchell (1989). Mutualistic interactions provide a fitness benefit to both organisms. In parasitic interactions, one partner benefits, and the other experiences reduced fitness. In a competitive interaction, both parties experience a reduction in fitness. Benefit or detriment was determined by comparing the fitness of the plant and fungus for a given resource exchange strategy and nutrient uptake efficiency combination with their fitness when both plant and fungus did not exchange resources (when the resource exchange strategy was 0%, 0%) for that same uptake efficiency combination. In this way, we were able to identify the mutualism zone, competition zones, and parasitic zones for each interaction type landscape.

### Evolution of stable resource exchange strategies

Finally, we used our fitness model in an individual‐based evolution model (IBM) to simulate the evolution of resource exchange strategies separately for each of the 231 uptake efficiency combinations (see Methods S1: The individual‐based evolution model). During these simulations, the nutrient uptake efficiencies remained the same and the resource exchange strategy (*γ*, *ε*) underwent evolution. Simulations were run until they reached a stable resource exchange strategy or resulted in the extinction of one or both populations. In all cases, this occurred between 500 and 2000 generations. Evolution simulations terminated if all the individuals in one or both populations experienced no growth during that generation, which was our definition of extinction.

For each of the uptake efficiency combinations, we characterised the coupled fitness landscape as having either (1) a stable or semi‐stable resource exchange strategy within the mutualism zone; (2) a stable or semi‐stable resource exchange strategy across both the mutualism and parasitism zones; or (3) no stable resource exchange strategy present, with evolution simulations ending in mutual or single extinction. Stable resource exchange strategies in other interaction type zones were not observed. A resource exchange area was defined to be stable or semi‐stable if at least one of 36 evolution simulations that were started at the center of it remained in the resource exchange strategy area for at least 500 generations (see Methods S1: Finding stable resource exchange strategies, Testing putative stable resource exchange strategies, Characterisation of stable resource exchange strategy areas, Characterisation of stability of stable and semi‐stable resource exchange strategies, and Finding the maximum fitness in the stable resource exchange strategy areas for details).

See ‘Methods S1: Overview of R code’ for details of the R code used and Notes S1 for all R code used.

## Results

### Stable mutualism

A stable and mutually beneficial resource exchange strategy evolved in many evolution simulations. Of the 231 coupled fitness landscapes tested, 66% had an evolutionarily stable resource exchange strategy area in the mutualism zone, whereas 25% of nutrient uptake efficiency combinations did not have any stable resource exchange strategies (see Fig. S1). In most cases, once the resource exchange strategy evolved to this area, it did not depart from it.

**Fig. 2:**
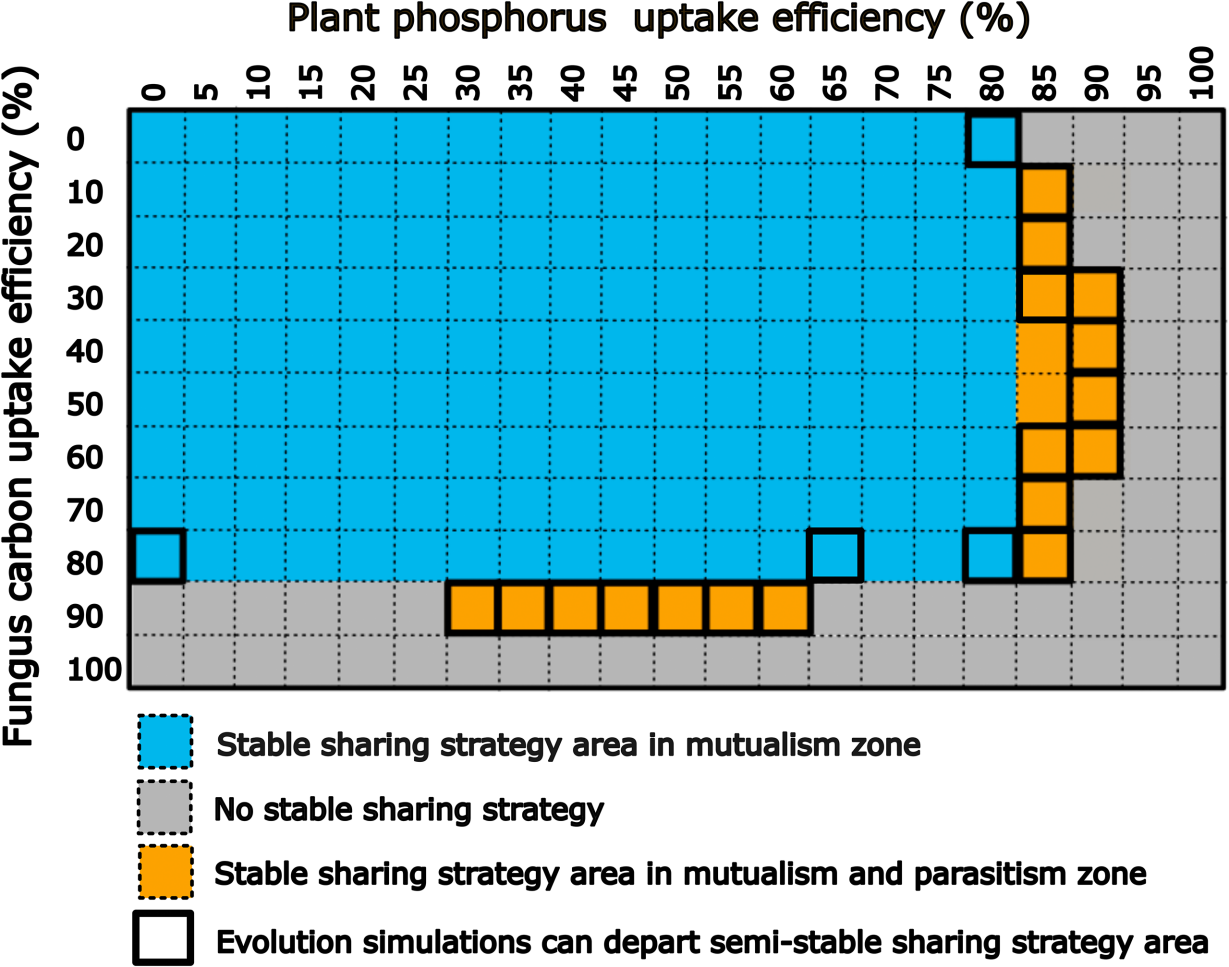
Interaction type and stability of the stable or semi‐stable resource exchange strategy areas for each combination of plant phosphorus (P) uptake efficiency and fungus carbon (C) uptake efficiency. The color indicates the interaction type present in the stable or semi‐stable resource exchange strategy area: blue shows that the stable resource exchange strategy area was located within the mutualism zone and orange shows that the area existed within both the mutualism and parasitic zones. Grey shows the nutrient uptake efficiency combinations that had no stable or semi‐stable resource exchange strategies. Bolded squares denote nutrient uptake efficiency combinations which had semi‐stable resource exchange strategy areas (see Supporting Information Methods S1 for details).

No nutrient uptake efficiency combination had an evolutionary stable resource exchange strategy area located only in the competition or parasitism zones. A single stable resource exchange strategy area in the mutualism zone was always present on the coupled fitness landscapes where both fungus C uptake efficiency and plant P uptake efficiency were 75% or lower (Fig. 2). The percent of initial resource exchange strategies that evolved to a stable resource exchange strategy area decreased as nutrient uptake efficiencies increased, once nonviable initial resource exchange strategies were removed from the total (Fig. S2).

**Fig. 3:**
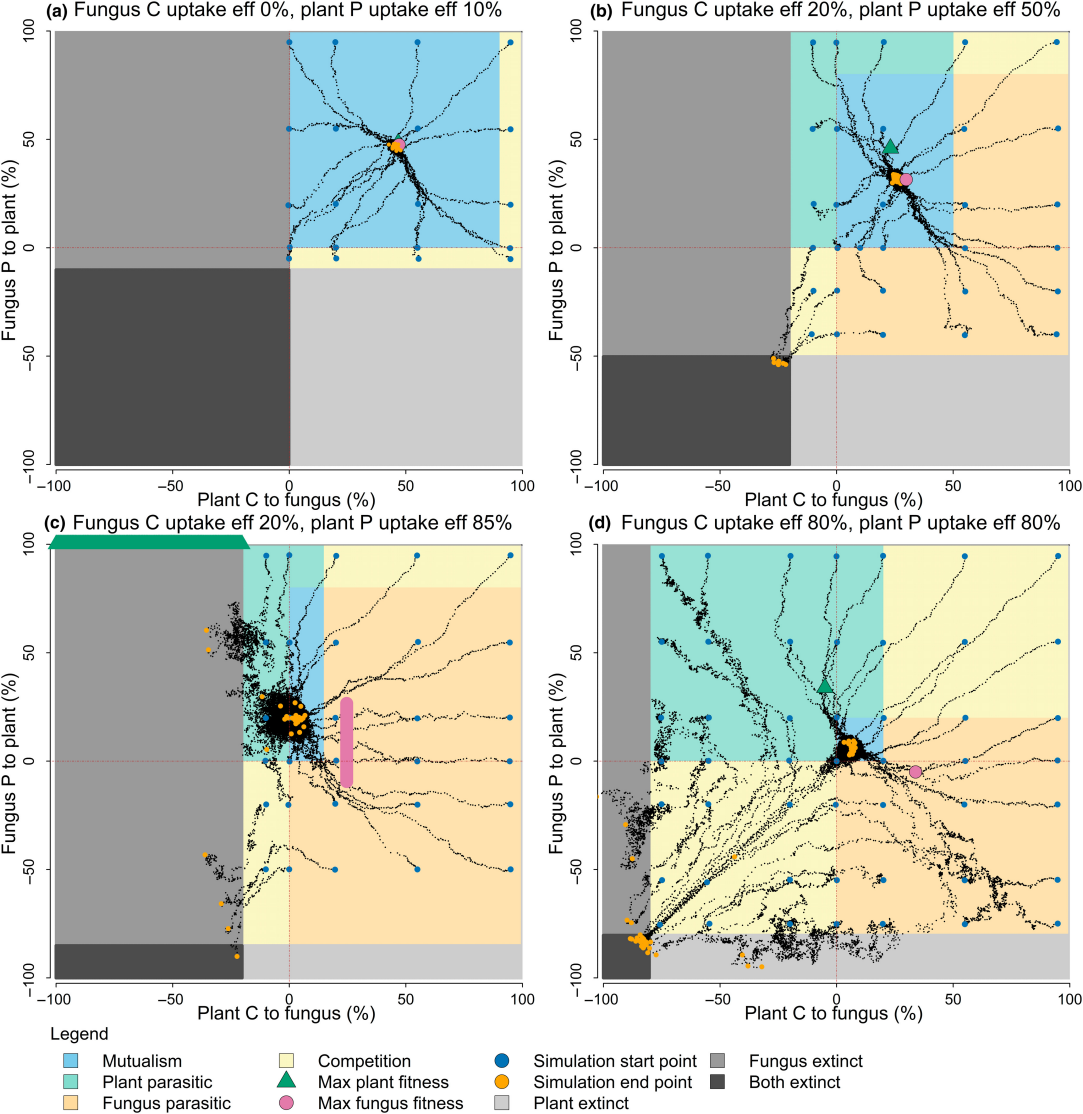
Evolutionary trajectories of the plant–fungus resource exchange strategy over 2000 generations in four nutrient uptake efficiency combinations. Each panel represents a different level of dependence that the organisms have on their partner for their nonspecialised resource (phosphorus (P) for the plant and carbon (c) for the fungus). (a) Both highly dependent: fungus C uptake efficiency of 0%, plant P uptake efficiency of 10%. (b) Both moderately but asymmetrically dependent: fungus C uptake efficiency 20%, plant P uptake efficiency 50%. (c) Fungus highly dependent, plant much less dependent: fungus C uptake efficiency 20%, plant P uptake efficiency 85%. (d) Both highly independent: fungus C uptake efficiency 80%, plant P uptake efficiency 80%. The dark blue circles and orange circles show the initial and final mean resource exchange strategies of the plant and fungus populations, respectively. Small black dots show the mean resource exchange strategy of the plant and fungus populations for a single generation of a simulation. The green triangles (or bars formed by overlapping triangles) and pink circles (or bars formed by overlapping circles) show the resource exchange strategy that results in the maximum plant and fungus fitness, respectively. Background colour indicates the interaction type of the organisms at that resource exchange strategy: mutualism (light blue), plant is parasitic (green), fungus is parasitic (orange), competition (yellow). Grey areas show where the resource exchange strategy results in the extinction of the plant, fungus, or both (light grey, medium grey, and dark grey, respectively). Note that many evolutionary trajectories ended at the same stable resource exchange strategy in the mutualism zone (a, b, d). Other evolutionary trajectories ended in extinction. Evolutionary trajectories could enter and move within the extinction zones as there were still some individuals in each population with viable resource exchange strategies.

The trajectory of evolution depended both on the fungus C uptake efficiency, plant P uptake efficiency, and the initial resource exchange strategy. Simulations that started in the competition zone within the first quadrant quickly left this zone and moved into one that benefited one or both organisms, often ending in a stable resource exchange strategy area in the mutualism zone (Fig. 3). Simulations that started in a parasitic zone could end either in the stable resource exchange strategy area or in the extinction zone of one or both organisms. On most coupled fitness landscapes, there was a region where, once an evolution simulation entered it, there was sufficient evolutionary pressure for evolution to quickly move to the stable resource exchange strategy in the mutualism zone. This region varied in size between nutrient uptake efficiency combinations and was located in and adjacent to the first quadrant, where both organisms exchange positive percentages of resources. Evolution simulations that started in the mutualism zone tended to remain in this zone and quickly find the stable resource exchange strategy area.

### Stable mutualistic‐parasitic resource exchange strategies

Another type of stable resource exchange strategy occurred when the stable area crossed the boundary between mutualism and a parasitic zone. Once the resource exchange strategy entered this area, it moved around within it, with the average interaction type switching between mutualism and parasitism (Fig. 3c). This reflects that the organisms' populations were made up of individuals experiencing mutualism and parasitism. A stable mutualistic‐parasitic resource exchange strategy was found on 8% of the coupled fitness landscapes and often occurred where one of the nutrient uptake efficiencies was above 80% (Figs 2, 3c).

### Transient parasitism

Many of the evolutionary trajectories that ended in stable mutualism crossed through a parasitism zone. These trajectories either started in a parasitism zone and entered the mutualism zone, or started in a competition zone and crossed through a parasitism zone before moving into the mutualism zone (Fig. 3). There were also examples of where the evolutionary trajectory moved through a parasitism zone and ended in an extinction zone (Fig. 3d). We refer to these as cases of transient parasitism.

### Semi‐stability

On most coupled fitness landscapes, once an evolutionary simulation reached the stable resource exchange strategy area, it did not leave. However, on 12% of the coupled fitness landscapes, when one of the nutrient uptake efficiencies was high (generally 85% or more), some simulations departed the stable resource exchange strategy area and ended in extinction. On these fitness landscapes, randomness took the simulation out of the stable resource exchange strategy area. The simulation then either returned to the stable resource exchange strategy area, moved into an extinction zone through random movement or entered the third quadrant competition zone and evolved to extinction (see Fig. S1b,c). The semi‐stable resource exchange strategy area became less stable as nutrient uptake efficiencies increased (see Fig. S3 for details).

### Single and mutual extinction

All evolution simulations either found a stable or semi‐stable resource exchange strategy area, unique to that coupled fitness landscape, or ended in extinction. When either nutrient uptake efficiency was 95 or 100%, no stable or semi‐stable resource exchange strategies were found (Fig. 2). On these coupled fitness landscapes, evolution resulted in the extinction of one or both organism populations. On most of the coupled fitness landscapes where stable resource exchange strategies existed, there were also simulations that ended in single or mutual extinction. Whether a simulation ended with a stable resource exchange strategy or in extinction depended on the initial resource exchange strategy of the simulation and where the evolutionary pathway moved on the coupled fitness landscapes. One type of pathway to extinction was when there was little evolutionary pressure on one or both organisms, and evolution entered the extinction zone of one organism through randomness (Fig. 3c). Another type of pathway to extinction was when evolutionary pressure drove both organisms to extinction.

Mutual extinction occurred when the evolutionary trajectory entered a region of negative feedback. This region was present on most coupled fitness landscapes in, and adjacent to, the third quadrant, where both organisms were taking resources. Once the evolutionary pathway entered it, the resource exchange strategy became increasingly negative for both organisms, eventually resulting in the extinction of one or both (in particular see Fig. 3d). This region increased in size as the nutrient uptake efficiencies increased.

### Maximum fitness, maximum resource received, and the stable and semi‐stable resource exchange areas

**Fig. 4:**
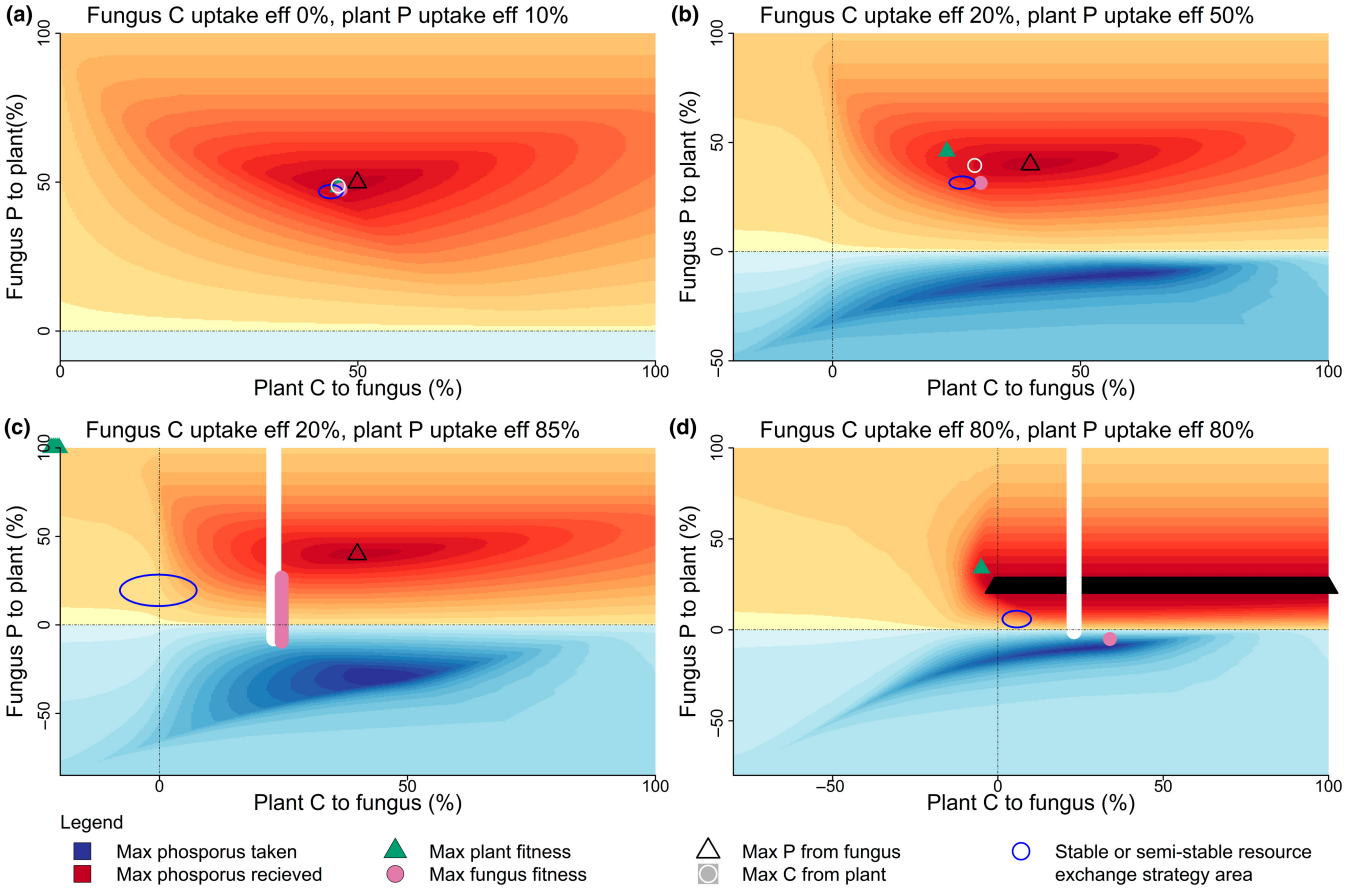
The total amount of phosphorus (P) given to the plant by the fungus across all viable resource exchange strategies. Each panel represents a different level of dependence that the organisms have on their partner for their nonspecialised resource: P for the plant and carbon (C) for the fungus. (a) Both highly dependent: fungus C uptake efficiency of 10%, plant P uptake efficiency of 10%. (b) Both moderately but asymmetrically dependent: fungus C uptake efficiency 20%, plant P uptake efficiency 50%. (c) Fungus highly dependent, plant much less dependent: fungus C uptake efficiency 10%, plant P uptake efficiency 80%. (d) Both highly independent: fungus C uptake efficiency 80%, plant P uptake efficiency 80%. Orange areas indicate the resource exchange strategies where the fungus gives P to the plant, with darker orange showing increasing amounts. Blue areas indicate where the fungus is taking P from the plant, with darker blue showing increasing amounts taken. The green triangle and pink circle show the resource exchange strategy that results in maximum plant and fungus fitness, respectively. The black triangle (or bar formed by overlapping triangles) and white circle (or bar formed by overlapping circles) show the strategies resulting in the maximum P given to the plant and the maximum C given to the fungus, respectively. The dark blue ellipses represent the stable resource exchange strategy area found by the individual‐based evolution simulations. Note that maximum organism fitness, maximum resource received, and the stable resource exchange strategy do not coincide.

The stable resource exchange strategy area generally did not coincide with the resource exchange strategy that resulted in an organism's maximum fitness. The resource exchange strategy resulting in an organism's maximum fitness only coincided with the stable resource exchange strategy when both nutrient uptake efficiencies were equal and low (Fig. S4a,b). As one or both organisms' nutrient uptake efficiencies increased, the resource exchange strategy for each organism's maximum fitness moved away from the stable resource exchange strategy and became further apart from each other (Fig. 4a–d).

The resource exchange strategy that resulted in the maximum amount of nonspecialised resource given to an organism was also not necessarily associated with the stable resource exchange strategy or the maximum fitness of an organism (Fig. 4). The stable resource exchange strategy only coincided with one or both maximum resource exchange strategies when both nutrient uptake efficiencies were below 20%. The maximum fitness of an organism only coincided with its maximum amount of nonspecialised resource received when either both nutrient uptake efficiencies were 0 or 10%, or when one nutrient uptake efficiency was below 10% and the other above 75% (Fig. 4c, see Fig. S4 for more details). We observed that the relationship between the plant and fungus nutrient uptake efficiencies and the stable and semi‐stable resource exchange strategy areas was nonlinear (see Figs S5–S7 for more detail).

## Discussion

### Stable mutualism from positive fitness feedback

In our model, stable mutualism was possible with only one‐to‐one fitness feedback as a stabilising mechanism. This contrasts with previous studies that suggest that mutual benefit alone is not enough for mutualism stability (Jones *et al*., 2015; Hammerstein & Noë, 2016). In fitness landscape regions where positive fitness feedback occurred, a change in the resource exchange strategy could benefit both organisms. The change allowed the receiver or donor to grow larger and acquire more resources, which meant that more resources could then be shared with their partner in the next time step, and so on. In these regions, a change in the resource exchange strategy that was detrimental to the plant would also be detrimental to the fungus and vice versa. This example of positive fitness feedback is identical to the definition of partner fidelity feedback proposed by Sachs *et al*. (2004). Other modelling experiments have also produced similar fitness feedback (de Mazancourt & Schwartz, 2010; Hilbe *et al*., 2018). For example, Hilbe *et al*. (2018) found that having repeated interactions and changing game yield allowed for populations to adapt quickly towards full cooperation. Our results suggest that when mutual benefit is the best strategy, mechanisms to counter the effects of cheating on the stability of mutualism may not be required, at least under certain circumstances.

### Real‐life observations of partner choice

Our model did not include proposed mutualism stabilising mechanisms that have been observed operating between plants and mycorrhizal fungi, such as proportional rewards, sanctions, and partner selection (Bever *et al*., 2009; Lekberg *et al*., 2010). Our results suggest that these mechanisms are not always necessary for the evolution of stable mutualism, but we do not argue that they are unimportant. A key advantage of showing that stable mutualism can evolve without these putative stabilising mechanisms is that many of these mechanisms involve complex signalling and physiological processes and thus must have evolved in a series of steps over generations. Simple, passive exchanges of nutrients, such as using one organism's waste products by another, have been cited as a possible precursor in the evolution of more active and sophisticated exchanges (Leimar & Hammerstein, 2010; Ruotsalainen *et al*., 2022). For instance, there is evidence that nutrients may simply diffuse out of intercellular hyphae of AMF, where they are present in high concentrations, into the surrounding fluid in the spaces between cortex cells (Ryan *et al*., 2003). It is likely that the collection of traits and mechanisms associated with today's mutualists have accumulated over time, and it may be that not all of them stabilise mutualism. Exploring how the presence, combination, and sequence of addition of other mechanisms affect mutualism stability will provide valuable insight into how these mechanisms support or impede mutualism evolution and stability.

### Cheating and the mutualism paradox

The search for mutualism stabilising mechanisms is motivated by the mutualism paradox, which states that mutualisms should be evolutionarily unstable as organisms will gain a fitness advantage by cheating, which will lead to mutualism breakdown. Here, we use the relative‐fitness‐based definition of cheating proposed by Jones *et al*. (2015) that ‘cheating can be defined as a change that increases the fitness of the actor above average fitness and decreases the fitness of the partner below average partner fitness.’ Cheating is defined as only possible in a context where mutualistic interactions exist. There were many instances in our evolution simulations where it was not possible for a change in the resource exchange strategy to produce an outcome where one organism benefited, while the other suffered a fitness loss. Therefore, in these regions cheating was not possible. However, in the study of mutualisms, cheating is often considered an ever‐present threat to mutualism.

Several prominent trade mutualism models assume that the provision of a commodity from one actor to another is always a cost to the first actor (Ferriere *et al*., 2002; Neuhauser & Fargione, 2004; Weyl *et al*., 2010; Cropp & Norbury, 2018). This automatically incorporates the assumption of cheating that underlies the mutualism paradox into the models in an omnipresent way, preventing these models from exploring parameter spaces where the assumption of cheating is false. The authors of these models then reason that actors will minimise the amount of commodity they lose to trade to prevent fitness loss, which in turn causes a reduction of fitness in the other actor. In these models, one actor must be cheating the other, since as one's fitness increases, the other decreases. However, supplying a certain commodity would only be a fitness cost if the actor was potentially limited by it.

The assumption that supplying C to a mutualistic fungus is always a cost to the plant has been previously challenged (Corrêa *et al*., 2012; Walder & Van Der Heijden, 2015; Prescott *et al*., 2020). If the plant can indeed supply the fungus with C without a loss of fitness, then both the plant and mycorrhizal fungus can simultaneously benefit from the resource trade. When this occurs, the premise of the mutualism paradox is not met. Assuming that the underlying premise of the mutualism paradox is always true when it may not be could lead to erroneous model outcomes. This highlights the importance of determining under what conditions actions would benefit one organism but be detrimental to their partner.

It should be noted that not all previous plant‐mycorrhizal trade models have imposed the condition of omnipresent cheating that is the premise of the mutualism paradox. These exceptions include the resource exchange model developed by Schwartz & Hoeksema (1998) and subsequent extensions of it (Hoeksema & Schwartz, 2003; de Mazancourt & Schwartz, 2010) and other original models (Hoeksema & Kummel, 2003; Kummel & Salant, 2006; Landis & Fraser, 2008; Grman *et al*., 2012; Verbruggen *et al*., 2012; Franklin *et al*., 2014; Henriksson *et al*., 2021; Shao *et al*., 2023). However, these models also did not include fitness feedback between the plant and mycorrhizal fungus, and as such, they were not suited to assessing whether mutualism could be evolutionarily stable with only fitness feedback.

### Darwinian extinction, cheating, and the mutualism paradox

In our model, cheating was most obvious when evolution entered the lower left competition zone. The fitness benefits of the resource exchange strategies in this area may seem counterintuitive, as organisms are taking their specialised resource, which is not limiting to, or contributing to, their growth. However, this resource is limiting to their partner. By taking it from their partner, that partner has reduced growth and a reduced ability to take the first organism's limiting resource. In other words, there is an advantage to the organism damaging their partner because their partner is damaging them. This meets the definition of cheating provided above, and so we can say that the assumption of cheating that underlies the mutualism paradox was present in this region.

As predicted by the logic of the mutualism paradox, when the evolutionary trajectory entered the lower left competition zone, the plant and mycorrhizal fungus populations quickly evolved to extinction. Causing more damage to one's partner gives a short‐term immediate fitness advantage compared to those doing less damage, but it results in long‐term extinction due to the ever‐escalating feedback loop of mutual damage. This is consistent with the concept of Darwinian extinction. In Darwinian extinction, phenotypes with higher relative fitness are selected for, but these phenotypes produce changes in the abiotic and biotic environment that decrease the mean fitness of the population and can even result in the extinction of the population (Webb & Travis, 2003) (also see Flintham *et al*., 2023). Darwinian extinction was present in most nutrient uptake efficiency combinations. However, it is possible that incorporating one or more of the proposed mechanisms for mutualism stability, such as partner selection or sanctions, may result in a different outcome.

### The stress gradient hypothesis, prevalence of mutualism, and parasitism

The results of our model are reminiscent of Bertness & Callaway's (1994) stress gradient hypothesis, which predicts that beneficial interspecies interactions will be more common in highly stressful conditions. As lower nutrient uptake efficiencies in our model can represent lower resource availability and thus more stress, our results are consistent with the stress gradient hypothesis. Mutualism became less stable and the mutualism zone smaller as nutrient uptake efficiencies increased. However, in real‐life situations, low resource availability is just one stress of many that organisms can experience.

In reality, mycorrhizal associations are often suppressed in high P environments compared to moderate P environments, which appears to parallel the stress gradient hypothesis. However, in extremely low P environments, mycorrhizas do not improve plant P uptake (Albornoz *et al*., 2021). In these environments, plants can be nonmycorrhizal and use alternate P acquisition strategies, such as cluster roots (Lambers *et al*., 2015). Viewed from this perspective, evidence of previous trade mutualism breakdown between plants and mycorrhizal fungi is consistent with the general results of our model. Further complications arise when we consider the other services, such as pathogen defense, that mycorrhizas can provide plant hosts (Albornoz *et al*., 2021).

In our model, parasitism only occurred when one or both nutrient uptake efficiencies were above zero. In these simulations, an organism being parasitised was able to tolerate losing both C and P and could still reproduce. If the organism being parasitised had a nutrient uptake efficiency of 0%, the attempt at parasitism would result in the death of the partner. It may be that beneficial interactions are more common in high‐stress environments because the non‐beneficial interactions have caused the extinction of one or both interacting species. Further work is needed to investigate the interactions between these factors before more concrete conclusions can be drawn.

### The evolution of mycorrhizal fungi

There is apparent extinction and emergence of new ectomycorrhizal communities through evolutionary time (Ryberg *et al*., 2022), in contrast to AMF, which form a single coherent lineage within the subphylum Glomeromycotina (Brundrett, 2002). This is paralleled in our model as the stability of mutualism decreased as nutrient uptake efficiencies increased. While it is unclear how the ancestors of Glomeromycotina acquired C before partnering with plants, it is likely that EMF ancestors were capable of saprotrophic activity when they first formed mycorrhizal associations (Miyauchi *et al*., 2020). The ability of ancestral EMF to source their own C may have increased their risk of mutualism breakdown, while the early loss of this ability in AMF may have resulted in a more stable mutualism early on. As the saprotrophic ability of ancestral EMF weakened through gene loss, their partnership with plants may have stabilised.

Unlike AMF and EMF, OMF and ErMF have robust complements of saprotrophic and mycorrhizal genes (Genre *et al*., 2020). Some species of orchid are dependent on their mycorrhizal fungal partners for C for their whole lives, while others only receive C during early stages of development. Interestingly, genera of OMF have differing abilities to access soil nitrate and nitrite (Albornoz *et al*., 2021). This raises the question of whether the orchid is becoming the specialised N supplier. Ericoid mycorrhizal fungi, on the other hand, have a dual saprotrophic and mycorrhizal lifestyle (Martino *et al*., 2018). Our model would predict their trade mutualism to be unstable. However, we should remember that mycorrhizal fungi provide services beyond mineral supply to their plant hosts (Albornoz *et al*., 2021), and more layers of complexity will need to be added to our model to capture these dynamics.

### Assuming interaction type via resource transfer

When investigating mutualisms, it is common practice to use indirect measurements to classify interspecific interactions, especially in field studies (Johnson *et al*., 1997). It is sometimes assumed that a reduction in nutrient supply to a partner indicates a reduction in mutualistic activity, a switch to parasitism, or an imminent breakdown in mutualism (Bennett & Groten, 2022). However, our results show that when Liebig's law of the minimum is assumed, the reduction in nutrient supply from a partner does not necessarily result in the reduction of fitness in the receiving organism. This means that the aforementioned assumption would not hold. On many points of our coupled fitness landscapes, there are areas where the reduction in the percent of nutrient supplied to a partner resulted in an increase in the fitness of the partner. This was most evident in the competition zone in the first quadrant of the coupled fitness landscapes.

Similarly, many models of interspecific interactions assume that maximising resources received or minimising resources given will maximise fitness. In our model, if we consider the total amount of nutrients supplied to a partner, we found that maximum fitness only coincides with maximum nutrient received when the organism is highly dependent on its partner for that nutrient. This highlights problems with inferring fitness outcomes through changes in nutrient exchange, as reviewed previously by Jones *et al*. (2015). While direct measurements of fitness are difficult to obtain in the field, our results support the conclusion that relying on changes in nutrient exchange to determine fitness effects and interaction type may lead to erroneous outcomes.

### Model limitations and future questions

A key aspect of our model was that, within their lifetimes, the individual plant and fungus are locked into repeated interactions and fitness feedback with one another. This seems to be a key factor in achieving stable mutualism in our model. Yet, in real life, plants can form mycorrhizal associations with multiple individual fungi at a time, and mycorrhizal fungi can form associations with multiple plants. It is possible that including multiple partners could destabilise the stable mutualism in our model or allow for more stable parasitism, as each individual's fitness could be less tightly linked to its host's fitness.

The plant and fungus in our model also did not have the ability to disengage from one another if the interaction was harmful to them. Various forms of partner choice have been observed operating between plants and mycorrhizal fungi (Bever *et al*., 2009; Lekberg *et al*., 2010). Including the ability to abandon harmful partners may have prevented the Darwinian extinction that we observed in our model.

Another component of our model was that the plant and fungus life span was made up of 10 interactions. This raises the question of how many repeat interactions are necessary for mutualism to stabilise. Having more or less timesteps in the growth model may change the evolutionary trajectory outcomes. Having an environment where resources were limited or patchy would add a level of realism that is currently lacking from our model. Future studies should explore these questions to better understand the subtleties of fitness feedback.

### Conclusion

We conclude that repeated interactions between individuals of mutual benefit alone are sufficient to lead to the evolution of stable mutualism between plants and fungi under certain circumstances, without the need for additional mutualism stabilising mechanisms. Furthermore, we show that cheating does not necessarily represent an omnipresent threat to mutualism stability, as deviation from mutually beneficial exchanges may not increase an organism's fitness. The principal novel aspect of our new fitness model is that it has produced outcomes, such as transitions in interaction types, stable mutualism, and stable mutualism–parasitism, which previous models have been unable to capture without the addition of specific terms designed to produce such events. Future research should explore how the addition of mechanisms associated with mutualism affects the evolution and stability of mutualism and extinction pathways. Our model is a baseline model for the development of more complex and realistic models of real‐life plant‐microbe mutualisms and other close ecological partnerships with repeat interactions.

## Competing interests

None declared.

## Author contributions

The original code for the growth model, fitness landscapes, and IBM was written by MR. The original code for the interaction landscapes was written by SVG. All code was further developed and refined by SVG. Simulations and analysis were conducted by SVG, with input from MR. Knowledge of plants and mycorrhizal fungi was provided by MHR and FEA. Project guidance was provided by MR, FEA, and MHR. All authors contributed to the interpretation of results and conceptual input. Writing was led by SVG, with contributions from all authors. MR, MHR, and FEA provided restraint on sojourns into abstract mathematics and theory.

## Disclaimer

The New Phytologist Foundation remains neutral with regard to jurisdictional claims in maps and in any institutional affiliations.

## Supporting information


**Fig. S1** Examples of nutrient uptake efficiency combinations with stable resource exchange strategies, semi‐stable strategies, and no stable resource exchange strategies.
**Fig. S2** Percent of simulations ending in the stable resource exchange strategy area.
**Fig. S3** Mean percent of simulations departing from the stable resource exchange area.
**Fig. S4** Total P given to plant and total C given to fungus for fungus C uptake efficiency (fCeff) 10% plant P uptake efficiency (pPeff) 10%, and fCeff 80% pPeff 80%.
**Fig. S5** The nutrient uptake efficiency of the partner at the organism's maximum fitness at the stable resource exchange strategy for a fixed value of the organism's nutrient uptake efficiency.
**Fig. S6** Plant and fungus fitness and the amount of nonspecialised resource shared at the stable resource exchange strategy found by the individual‐based evolution model (IBM).
**Fig. S7** Plant and fungus fitness at the stable resource exchange strategy found by the individual‐based evolution model (IBM).

**Methods S1** A document containing the details of the experiments and analysis.

**Notes S1** An R file containing all code needed to replicate the experiments.
Please note: Wiley is not responsible for the content or functionality of any Supporting Information supplied by the authors. Any queries (other than missing material) should be directed to the *New Phytologist* Central Office.

## Data Availability

All data used in this project were generated using the R code available in the Supporting Information (Notes SI).
