# Supplementary material for: A simple plant–mycorrhizal fungal resource trade co‐evolution model explains mutualism stability, extinction and transitory parasitism via fitness feedback
Source: New Phytol. 2025 Sep 12;248(3):1429–41. doi: 10.1111/nph.70540 (PMC12489293; doi:10.1111/nph.70540)
Supplement: Supplementary file 1 — Fig. S1 Examples of nutrient uptake efficiency combinations with stable resource exchange strategies, semi‐stable strategies, and no stable resource exchange strategies. Fig. S2 Percent of simulations ending in the stable resource exchange strategy area. Fig. S3 Mean percent of simulations departing from the stable resource exchange area. Fig. S4 Total P given to plant and total C given to fungus for fungus C uptake efficiency (fCeff) 10% plant P uptake efficiency (pPeff) 10%, and fCeff 80% pPeff 80%. Fig. S5 The nutrient uptake efficiency of the partner at the organism's maximum fitness at the stable resource exchange strategy for a fixed value of the organism's nutrient uptake efficiency. Fig. S6 Plant and fungus fitness and the amount of nonspecialised resource shared at the stable resource exchange strategy found by the individual‐based evolution model (IBM). Fig. S7 Plant and fungus fitness at the stable resource exchange strategy found by the individual‐based evolution model (IBM). [file NPH-248-1429-s001.pdf]

Supplementary figures

New Phytologist Supporting Information

Article title: A simple plant-mycorrhizal fungal resource trade co-evolution model explains mutualism stability, extinction, and transitory parasitism via fitness feedback

Authors: Sally V. Grasso, Megan H. Ryan, Felipe E. Albornoz, Michael Renton

Article acceptance date: 8 August 2025

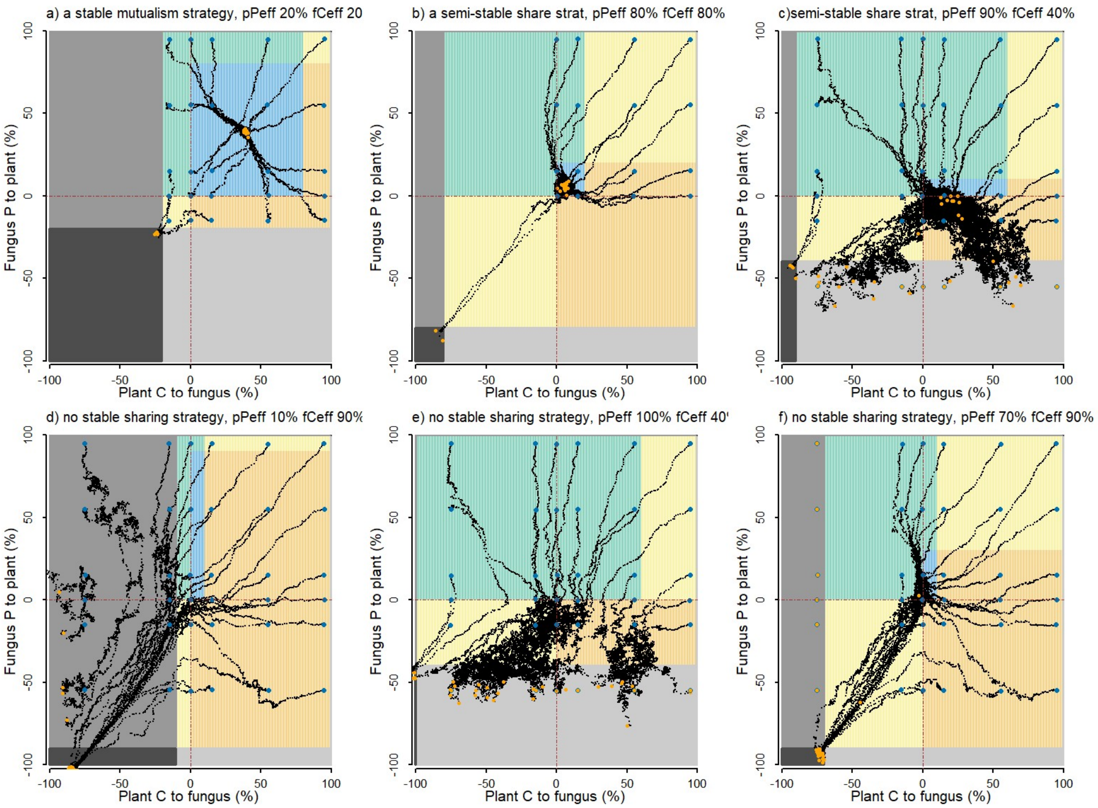

Supplementary Figure S1. Examples of nutrient uptake efficiency combinations with stable resource exchange strategies, semi-stable strategies, and no stable resource exchange strategies. The dark blue circles and orange circles show the mean resource exchange strategy of the plant and fungus populations of the initial and final generations, respectively. Small black dots show the mean resource exchange strategy of both plant and fungus populations for a single generation of a simulation. Background colour indicates the interaction type between the organisms at that resource exchange strategy: mutualism (light blue), plant is parasitic (green), fungus is parasitic (orange), competition (yellow). Grey areas show where the resource exchange strategy results in the extinction of the plant, fungus, or both (light grey, medium grey, and dark grey, respectively). pPeff = plant phosphorus uptake efficiency, fCeff = fungus carbon uptake efficiency. To allow for visual clarity, not all simulations run for each nutrient uptake efficiency combination are shown.

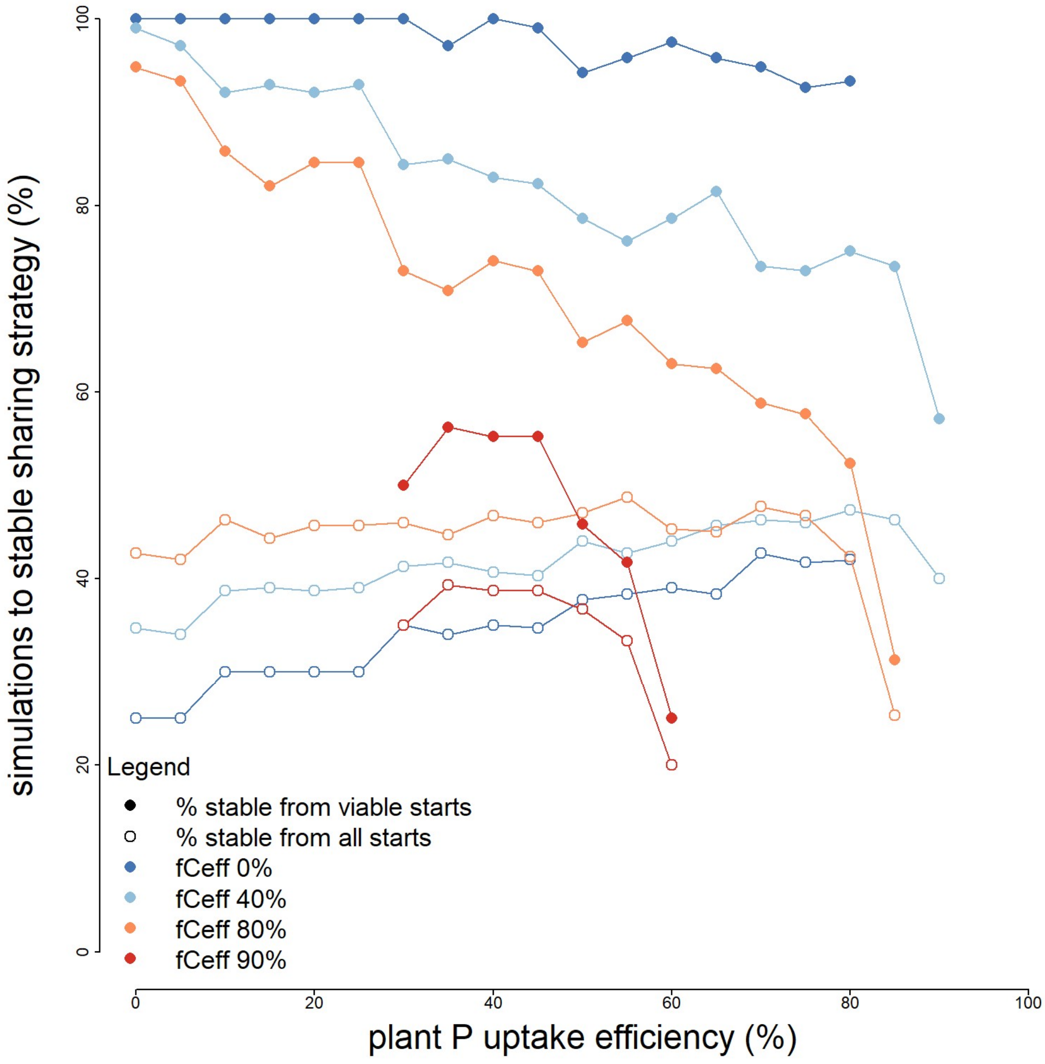

Supplementary figure S2. Percent of simulations ending in the stable resource exchange strategy area. Closed circles indicate the percent of simulations originating from viable resource exchange strategies. Open circles indicate the percent of all simulations regardless of the viability of the initial resource exchange strategy. On each coupled fitness landscape the IBM was run from 100 unique initial resource exchange strategies with 3 reps. Each simulation was run until it reached the stable resource exchange strategy or ended in extinction.

fCeff (%)	pPeff (%)	mean % of departures	sd
0	80	0.67	1.15
10	85	6.00	4.00
20	85	2.00	2.00
30	85	2.00	2.00
30	90	20.67	4.62
40	90	12.67	4.16
50	90	20.67	2.31
60	85	2.67	1.15
60	90	82.00	0.00
70	85	16.00	5.29
80	85	70.67	5.03
80	0	0.67	1.15
80	65	0.67	1.15
80	80	4.67	3.06
90	35	16.67	4.62
90	45	18.67	6.43
90	55	46.67	5.77
90	30	30.67	7.57
90	40	11.33	2.31
90	50	26.00	5.29
90	60	76.67	4.16

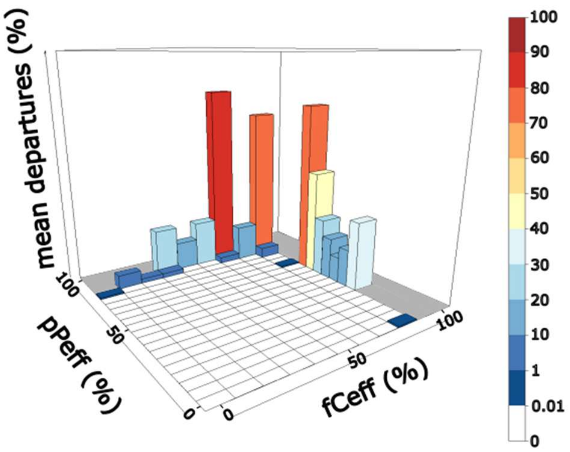

Supplementary Figure S3. Mean percent of simulations departing from stable resource exchange area. IBM was run from centre of the stable resource exchange area for 500 generations with 50 x 3 reps. pPeff = plant phosphorus uptake efficiency, fCeff = fungus carbon uptake efficiency. The stability of the stable resource exchange area in nutrient uptake efficiency combination that correspond to the grey squares of Fig 2. were not tested, as these coupled fitness landscapes did not contain any stable or semi-stable resource exchange areas.

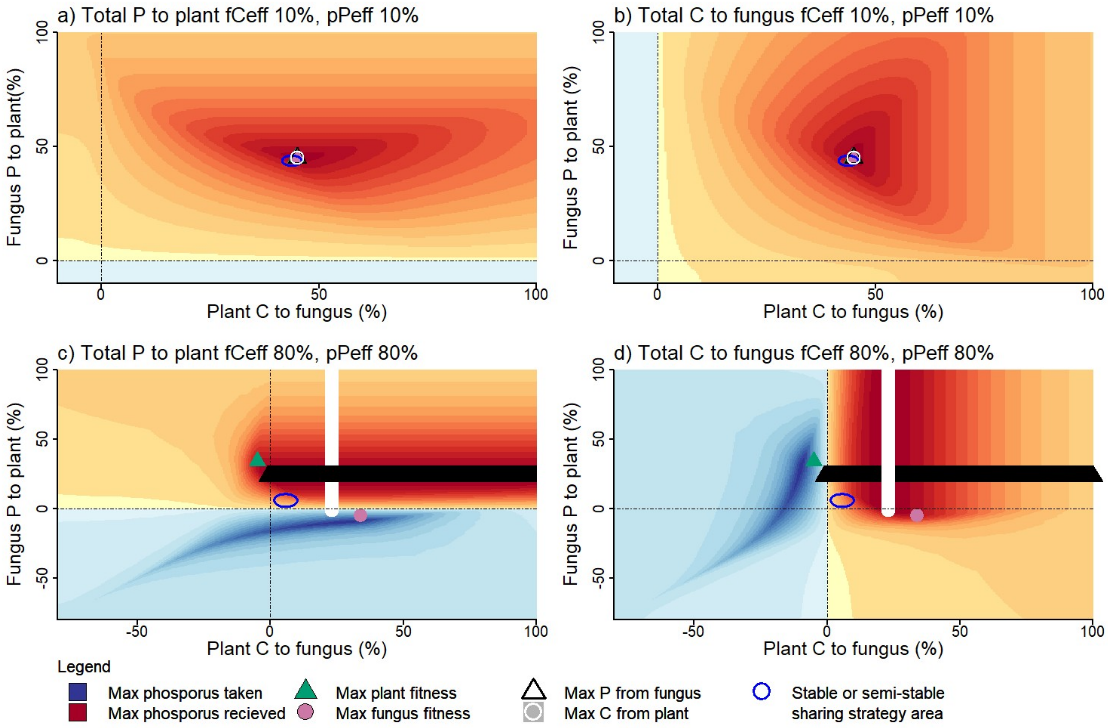

Supplementary figure S4. Total P given to plant and total C given to fungus for fungus C uptake efficiency (fCeff) 10% plant P uptake efficiency (pPeff) 10%, and fCeff 80% pPeff 80%. Orange areas indicate the resource exchange strategies where the partner gives the resource to the organism, with darker orange showing increasing amounts given. Blue areas indicate where the partner is taking resource from the organism, with darker blue showing increasing amounts taken. The green triangle and pink circle show the resource-resource exchange strategy that results in maximum plant and fungus fitness, respectively. The black triangle (or bar formed by overlapping triangles) and white circle (or bar formed by overlapping circles) show the strategies resulting in the maximum P given to the plant and the maximum C given to the fungus, respectively. The dark blue ellipses represent the stable resource exchange strategy area found by the individual-based evolution simulations. Note that maximum organism fitness, maximum resource received, and the stable resource exchange strategy only coincide in panels (a) and (b), where the plant and fungus are highly and symmetrically dependent.

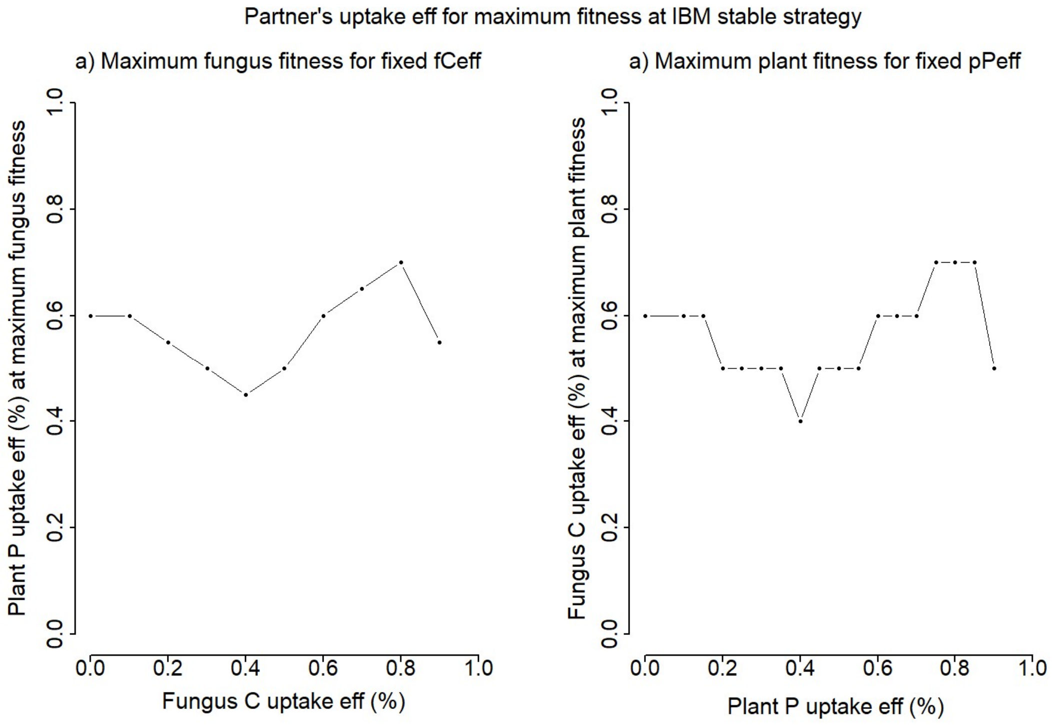

Supplementary figure 5. The nutrient uptake efficiency of the partner at the organism's maximum fitness at the stable resource exchange strategy for a fixed value of the organism's nutrient uptake efficiency. For example, in a) when fungus C uptake efficiency =0% the highest fungus fitness at any stable resource exchange strategy occurred when plant P uptake efficiency = 60%. pPeff = plant phosphorus uptake efficiency, fCeff = fungus carbon uptake efficiency.

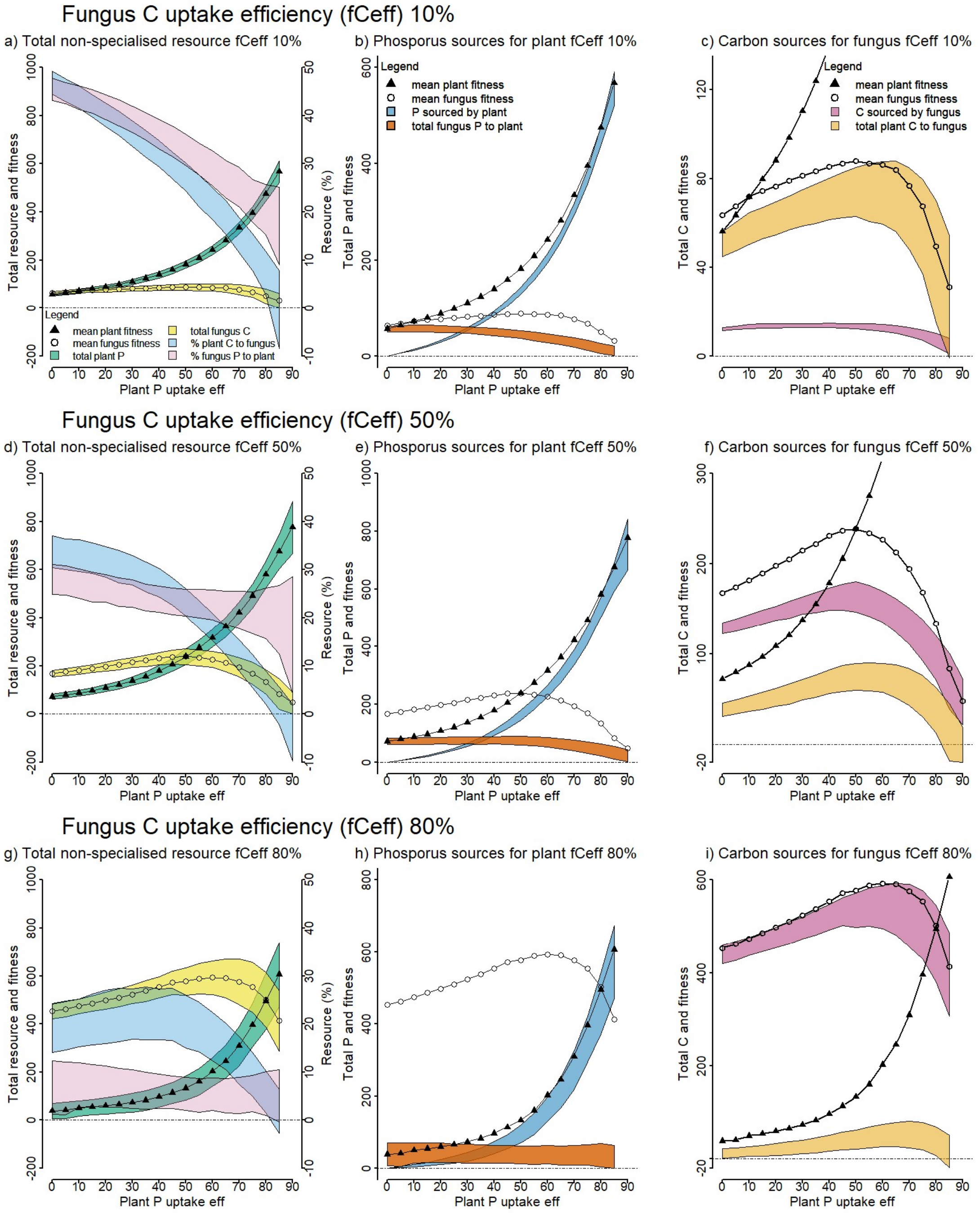

Supplementary Figure 6. Plant and fungus fitness and the amount of non-specialised resource shared at the stable resource exchange strategy found by the individual-based evolution model (IBM). This figure shows how fitness, total non-specialised resource, and the percentage of non-specialised resource changed at the IBM stable resource exchange strategy as plant phosphorus uptake efficiency (plant P uptake efficiency) increased for three fixed values of fungus carbon uptake efficiency

(fungus C uptake efficiency): 10%, 50%, and 80% in the upper, middle, and lower horizontal panels, respectively. pPeff = plant phosphorus uptake efficiency, fCeff = fungus carbon uptake efficiency.

The left vertical panels show the total acquired non-specialised resource (plant phosphorus in green and fungus carbon in yellow), the mean fitness of each organism (black triangles for plants and open circles for fungi), and the percentage of specialised resource given to their partner (light blue for the percentage of plant carbon given to fungus and light pink for the percentage of fungus phosphorus given to plants). The middle vertical panels show each organism's mean fitness, and the sources of the total phosphorus acquired by the plant (phosphorus taken up by the plant in red and phosphorus given to the plant by the fungus in dark blue). The right vertical panels show each organism's mean fitness, and the sources of the total carbon acquired by the fungus (carbon taken up by the fungus in dark pink and carbon given by the plant in orange). The y-axis scale differs among the panels. Values at plant P uptake efficiency = 90% are only shown in the middle horizontal panels because no IBM stable resource exchange strategies were present at this plant P uptake efficiency when fungus C uptake efficiency was 10% or 80%. Note that the total amount of non-specialised resource acquired at the stable resource exchange strategy was most heavily influenced by the organism's own nutrient uptake efficiency, with resource uptake by the organisms tending to increase as the organism's nutrient uptake efficiency increased and the amount given by the partner decreased. However, it was also influenced by the partner's nutrient uptake efficiency, as seen in the right vertical panels, where carbon taken up by the fungus increased and then decreased as plant P uptake efficiency increased. The proximity of each organism's mean fitness to the sources of their own non-specialised resource indicates the organism's dependence on their partner for their non-specialised resource.

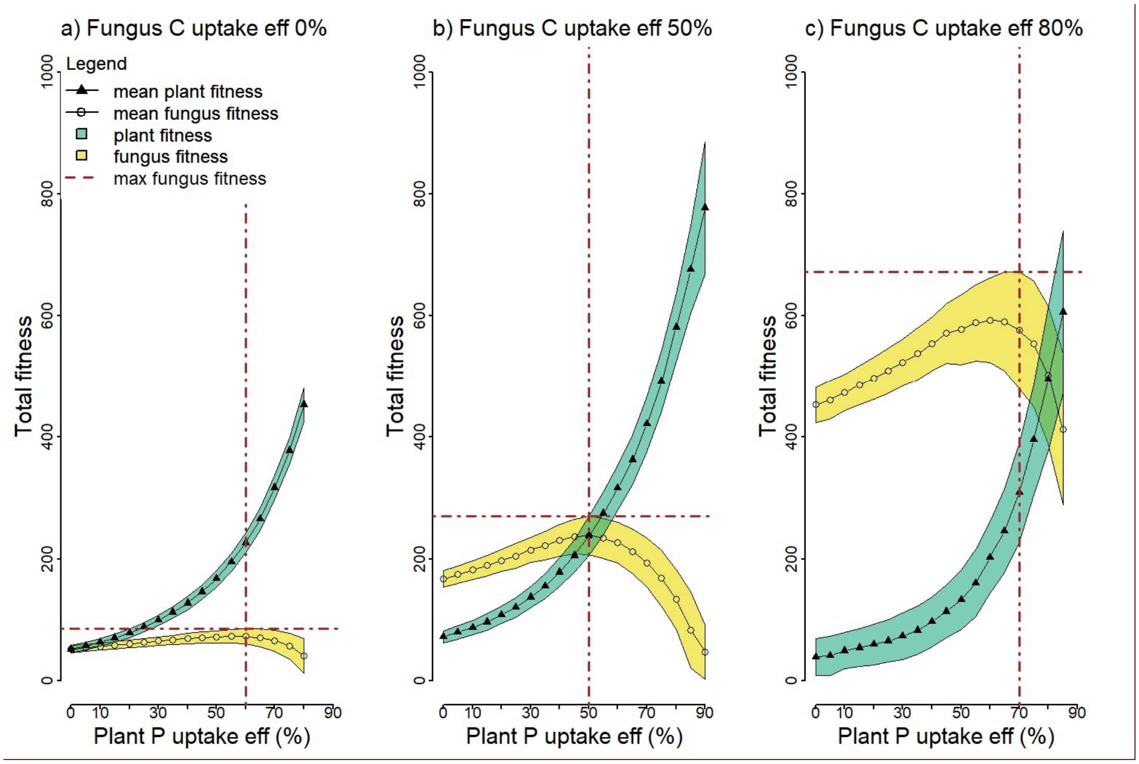

Supplementary figure 7. Plant and fungus fitness at the stable resource exchange strategy found by the individual-based evolution model (IBM). This Figure shows how the organisms' fitness changed at the IBM stable resource exchange strategy as plant phosphorus (P) uptake efficiency increased for three fixed values of fungus carbon (C) uptake efficiency: (a) 10%, (b) 50%, and (c) 80%. Green shows the range of plant fitness at the stable resource exchange strategy, and yellow the range of fungus fitness. The median fitness of each organism is shown by black triangles for plants and open circles for fungi. The dashed brown line shows the maximum fungus fitness and the plant P uptake efficiency it occurred at.

Comment on Fig. S6 and Fig. S7.

An organism's fitness at the stable resource exchange strategy was mainly influenced by its own nutrient uptake efficiency but was also affected by their partner's nutrient uptake efficiency. The general trend was for the organism's fitness to increase as its nutrient uptake efficiency for its non-specialised resource increased. However, when the first organism's nutrient uptake efficiency was fixed and its partner's was increasing, we observed a more complex interaction. The fitness of the first organism increased and then decreased as its partner's nutrient

uptake efficiency increased (Fig. 5a-c). The plant P uptake efficiency at which the maximum fungus fitness occurred was not linearly correlated with the fungus C uptake efficiency. The lowest plant P uptake efficiency (45%) at the fungus maximum occurred when the fungus C uptake efficiency was 40%, and the maximum plant P uptake efficiency (70%) at the fungus maximum occurred when the fungus C uptake efficiency was 80% (Fig. S5). See Supporting Figure S6 for details on how an increasing plant P uptake efficiency affects both organisms' acquisition of non-specialised resource, their fitness, and their dependence on their partner at the stable resource exchange strategy.
