# Supplementary material for: A simple plant–mycorrhizal fungal resource trade co‐evolution model explains mutualism stability, extinction and transitory parasitism via fitness feedback
Source: New Phytol. 2025 Sep 12;248(3):1429–41. doi: 10.1111/nph.70540 (PMC12489293; doi:10.1111/nph.70540)
Supplement: Supplementary file 2 — Methods S1 A document containing the details of the experiments and analysis. [file NPH-248-1429-s003.pdf]

New Phytologist Supporting Information

Article title: A simple plant-mycorrhizal fungal resource trade co-evolution model explains mutualism stability, extinction, and transitory parasitism via fitness feedback

Authors: Sally V. Grasso, Megan H. Ryan, Felipe E. Albornoz, Michael Renton

Article acceptance date: 8 August 2025

**Supplementary Methods S1**

S1.1 Description of mathematical equations

Eqn. 1

The underlying equations describing of our system are as follows

$$X_{n+1} = X_n + \min\begin{pmatrix}\alpha X_n + \varepsilon Y_n \,, \\ X_n(1-\gamma)\end{pmatrix}$$

$$Y_{n+1} = Y_n + \min\begin{pmatrix}Y_n(1-\varepsilon)\,, \\ \beta Y_n + \gamma X_n\end{pmatrix}$$

Eqn. 1

where: $X_n$ and $Y_n$ are respectively the plant and fungus biomass at timestep n; α is the plant P uptake efficiency; β is the fungus C uptake efficiency; γ the percentage of plant C given to the fungus; and ε is the percentage of fungus P given to the plant. The quantities α and β can take values anywhere within the range 0 and 1 inclusive, and γ and ε can take any values between –1 and 1 inclusive. The initial conditions at timestep n = 0 are $X_0 = Y_0 = 1$. These equations are coupled and recursive, where the growth of each organism is dependent on the other, and each time step builds on the one before. In instances where one organism ‘gives’ a negative amount it may attempt to take more resource than the other organism has, which results in an interaction outside of the bounds of these two equations. In such a case the first organism will take all the available resource from the second organism in that time step, but no more (see below equations). This condition can change between time steps, as $X_n$ and $Y_n$ change.

Eqn. S1

$$X_{n+1} = X_n + \min\begin{pmatrix} \alpha X_n + \varepsilon Y_n\,, \\ X_n + \ \beta Y_n \end{pmatrix}$$

$$Y_{n+1} = Y_n + \min\begin{pmatrix} Y_n(1-\varepsilon)\,, \\ 0 \end{pmatrix}$$

Eqn. S1

$$X_{n+1} = X_n + \min\begin{pmatrix} 0\,, \\ X_n(1-\gamma) \end{pmatrix}$$

$$Y_{n+1} = Y_n + \min\begin{pmatrix} Y_n + \ \alpha X_n, \\ \beta Y_n + \gamma X_n \end{pmatrix}$$

Eqn. S2

$$X_{n+1} = X_n + \min\begin{pmatrix} 0\,, \\ X_n + \ \beta Y_n \end{pmatrix}$$

$$Y_{n+1} = Y_n + \min\begin{pmatrix} Y_n + \ \alpha X_n\,, \\ 0 \end{pmatrix}$$

Eqn. S3

S1.2 The individual based evolution model

To simulate evolution, we used an individual based evolution model. In this model we had a population of mycorrhizal fungi and a population of plants. Each population had 100 individuals. In each generation each plant was randomly paired with one fungus. Their fitness was then calculated using the fitness model. This was done for all pairs. The next generation of 100 plants and 100 fungi was then selected using a weighted random selection, with the fitness score of the individual being the weighting. Replacement was allowed, meaning that an individual could have multiple

“offspring” in the next generation. The % plant C to fungus and % fungus P to plant inherited by the individual offspring was then randomly changed a small amount, positively or negatively. Then each plant was paired with a random fungus and so on. Reproduction can be considered to be asexual with a high mutation rate. For more detail see the IBM code in  Notes S1.R file.

S1.3 Finding stable resource exchange strategies

We set out to locate the stable points on each pair of coupled fitness landscapes using the IBM code. We started by locating the stable point on the coupled fitness landscapes with a plant P uptake efficiency of 0% by running the evolution algorithms for 2000 generations (gens) from multiple initial resource exchange conditions of the unique combinations of -75%, -55%, -35%, -15%,-5%, 0%, 5%,15%, 35%, 55%, 75%, 95% for % plant C to fungus and % fungus P to plant. The resource exchange strategies at the last generation were compared to see if the evolutionary pathways converged to a common point. This point was considered a putative stable resource exchange strategy.

We then used this stable point as the initial resource exchange conditions for the coupled fitness landscapes with the next increment of plant P uptake efficiency (plant P uptake efficiency = 5%). We ran the evolution models for 200 gens with 3 repeats (reps). The average final resource exchange strategy from these simulations were used as the initial resource exchange conditions for the landscapes with the next plant P uptake efficiency value of 10% and so on until the whole range of plant P uptake efficiency and fungus C uptake efficiency combinations had been covered.

The evolutionary pathways on the coupled fitness landscapes of fungus C uptake efficiency 90% plant P uptake efficiency 0% did not converge to a common point.  To find putative stable points for fungus C uptake efficiency 90% plant P uptake efficiency 5%, plant P uptake efficiency 10% etc. The stable point from fungus C uptake efficiency 80% plant P uptake efficiency X% was used as the initial resource exchange strategy.

Coupled fitness landscapes that appeared to have no stable points were further tested by running the IBM for 2000 gens from multiple initial resource exchange

strategies (unique combinations of -75%, -55%, -35%, -15%,-5%, 0%, 5%,15%, 35%, 55%, 75%, 95% for % plant C to fungus and % fungus P to plant).

Plots from each test were visually inspected for signs of a stable point where evolution pathways converged.

## S1.4 Testing putative stable strategies

We then started the evolution simulations at these putative stable points and ran them for 500 gens for 36 reps. We visually inspected evolution pathways and end points to assess whether the evolution simulations converged and remained around a single point or were moving at random. A nutrient uptake efficiency combination was determined to have a stable resource exchange strategy if no simulations departed the immediate area where the simulations started. If at least one simulation remained in the immediate area in the 500 gens, then the nutrient uptake efficiency combination was determined to have a semi-stable resource exchange strategy. If no simulations remained in the immediate area, it was determined that no stable or semi-stable resource exchange strategies existed for that nutrient uptake efficiency combination.

## S1.5 Characterisation of stable resource exchange strategy areas

To determine the size and location of the stable and semi-stable resource exchange areas for each nutrient uptake efficiency combination we ran another 36 simulations starting at the stable or semi-stable resource exchange strategy for 500 gens. After excluding simulations that ended in extinction or left the stable area, we took the average end point of the simulations and the average maximum and minimum resource exchange % for each simulation. The average end point was adopted as the centre of the stable resource exchange strategy area and average maximum and minimum resource exchange values used for the diameter of the resource exchange area. A worked example of this process is in “Finding, testing, and characterising Stable Resource Exchange Strategy Area: A worked example ” of Notes S1.R.

S1.6 Characterisation of the stability of stable and semi-stable resource exchange strategies

To determine the stability of the stable and semi-stable resource exchange strategy areas we recorded the number of times evolution simulations left the areas. Starting from the centre of the stable or semi-stable area we ran 50 simulations for 500 gens. We recorded the number of simulation end points that remained within two SD of the minimum and maximum resource exchange % of the stable, or semi-stable, resource exchange area.

We repeated this twice more and took the average and SD of the number of simulations that remained within the area.

S1.7 Finding the maximum fitness in the stable resource exchange strategy areas

As the location and value of plant and fungus maximum fitness on the entire coupled fitness landscapes were not intuitive it was decided that the stable resource exchange strategy areas should be rendered and searched to find their fitness maximums and ranges.

A couples fitness landscape was made of each stable, or semi-stable, resource exchange strategy areas of 500 equally spaced values of the maximum and minimum % plant C to fungus and % fungus P to plant values of the area using the fitness landscape code. The average fitness and range of fitness values or the plant and fungus were then found.

S1.8 Over-view of Notes S1.R

This file contains all of the R code needed to replicate these experiments. It can be run in R or in R studio and can be viewed in a text editor.

The code was developed in:

**RStudio 2024.09.1** Build 394 © 2009-2024 Posit Software, PBC.

RStudio 2024.09.1+394 "Cranberry Hibiscus" Release (a1fe401fc08c232d470278d1bc362d05d79753d9, 2024-11-03) for windows

Mozilla/5.0 (Windows NT 10.0; Win64; x64) AppleWebKit/537.36 (KHTML, like Gecko) RStudio/2024.09.1+394 Chrome/124.0.6367.243 Electron/30.4.0 Safari/537.36, Quarto 1.5.57

The contents of Notes S1.R are:


- S1.1 Fitness surface code [Line 1]
- S1.2 Interaction type code [Line 259]
- S1.3 Individual based evolution of resource sharing [Line 480]
- S1.4 Resources given and taken landscape code [Line 888]
- S1.5 Finding, testing, and characterising Stable Resource Exchange Strategy Area: A worked example. [Line 1251]


The ‘fitness surface code’ produces results that are used in ‘interaction type code’, which in turn, produces results that are used in the plotting function of ‘Individual based evolution of resource sharing’.

The ‘resources given and taken landscape code’ and ‘fitness surface code’ produce results that are used in the plotting function of ‘resources given and taken landscape code’. There are also data sets that are needed to plot points of interest (i.e. the stable resource exchange strategy areas) that need to be loaded.

The R code used in this project was made in and run on **RStudio 2024.09.1** Build 394 © 2009-2024 Posit Software, PBC.

RStudio 2024.09.1+394 "Cranberry Hibiscus" Release (a1fe401fc08c232d470278d1bc362d05d79753d9, 2024-11-03) for windows

Mozilla/5.0 (Windows NT 10.0; Win64; x64) AppleWebKit/537.36 (KHTML, like Gecko) RStudio/2024.09.1+394 Chrome/124.0.6367.243 Electron/30.4.0 Safari/537.36, Quarto 1.5.57
